# Effects of a WhatsApp-Delivered Education Intervention to Enhance Breast Cancer Knowledge in Women: Mixed-Methods Study

**DOI:** 10.2196/17430

**Published:** 2020-07-21

**Authors:** Antonio Augusto Claudio Pereira, Juliana Regina Destro, Marcelo Picinin Bernuci, Lucas França Garcia, Tiago Franklin Rodrigues Lucena

**Affiliations:** 1 Universidade Cesumar Maringá Brazil; 2 ICETI-Cesumar Institute of Science, Technology, and Innovation Maringá Brazil; 3 Departamento de Fundamentos da Educação Universidade Estadual de Maringá Maringá Brazil

**Keywords:** mHealth, WhatsApp, cancer education, breast cancer

## Abstract

**Background:**

Breast cancer is the leading cause of cancer-related death in the female population. Health education interventions based on the use of mobile technologies enable the development of health self-care skills and have emerged as alternative strategies for the control of breast cancer. In previous studies, WhatsApp has stood out as a useful tool in health education strategies; however, it has not yet been applied for breast cancer education.

**Objective:**

This study aimed to analyze the potential of WhatsApp as a health education tool used to improve women's knowledge on the risk reduction of breast cancer. It also aimed to understand how women feel sensitized within the WhatsApp group throughout the intervention and how they incorporate information posted to improve knowledge about early detection and risk reduction methods.

**Methods:**

The study involved a pre-post health educational intervention with 35 women (aged 45-69 years) included in a WhatsApp group to share information (audio, video, text, and images) over 3 weeks on the early detection and risk reduction of breast cancer. Data were collected through questionnaires on topics related to risk reduction, as well as qualitative content analysis of group interactions. Effectiveness and feasibility were analyzed through conversations and the comparison of the scores obtained in the questionnaires before and after the intervention.

**Results:**

A total of 293 messages were exchanged (moderator 120 and users 173). The average scores of the participants were 11.21 and 13.68 points before and after the educational intervention, respectively, with sufficient sample evidence that the difference was significant (*P*<.001). The intervention enabled women to improve their knowledge on all topics addressed, especially “myths and truths,” “incidence,” “clinical manifestations,” and “protective factors.” Some themes emerged from the interactions in the group, including group dynamics, general doubts, personal narratives, religious messages, daily news, and events.

**Conclusions:**

The use of groups for women in WhatsApp for health education purposes seems to be a viable alternative in strategies on breast cancer control, especially as it provides a space for the exchange of experiences and disinhibition. However, the need for a moderator to answer the questions and the constant distractions by members of the group represent important limitations that should be considered when improving this strategy.

## Introduction

Breast cancer represents the main cause of death by cancer among women around the world [1]. The mortality rates associated with this malignancy are higher in low- and middle-income countries owing to early diagnosis deficiencies and low access to treatment [2,3]. Mortality reduction in many developed countries has been occurring after the implementation of organized screening of precursor lesions associated with more effective therapies [4-6]. However, the adoption of such strategies requires high infrastructure and human resource investments, which are below the capacity of low- and middle-income countries [7,8].

Considering financial constraints, it is suggested that the most prudent action to be applied in cancer control is the guarantee of early diagnosis and access to treatment without delay [9,10]. Nevertheless, the limitations of population awareness regarding the relevance of early diagnosis and preventive behavior constitute major barriers to the effectiveness of these actions [11-13]. Educational interventions mediated by mobile technologies represent alternatives to be explored in the practice of health promotion for their convenience and ubiquity, and for minimizing the barriers of distance, cost, and time [14-16].

The wide availability and functionality of mobile devices have made them great tools for planning, executing, and evaluating health interventions [17,18]. Indeed, smartphones and their apps have been used to spread health information [19-22], including information for breast cancer care [23]. Evidence for individuals who use health self-management apps points to positive results [24-26] and reinforces the viability of using this technology in health promotion strategies.

Among the apps with the potential for use in health education interventions, WhatsApp (WhatsApp Inc) stands out [27,28]. It is an instant messaging app for smartphones that enables users to send voice, text, video, or location using the group communication feature [29]. The app offers additional social information to its users when compared with SMS text messaging. Contacts are able to see when other users are online and typing, as well as their last access. Moreover, it is the most downloaded app in the world [30], is free, and has a user-friendly interface, and it accounts for about 20% of total smartphone use [31].

Despite the popularity of WhatsApp and its promising potential in health education and health care strategies [27,32-34], only few studies have evaluated the effectiveness of using this app to convey health information for user empowerment purposes [33,35-38]. Therefore, this study aimed to provide evidence for the utility and effectiveness of this mobile health technology to improve women's knowledge regarding the risk reduction and early detection of breast cancer. The primary aim was to evaluate women's engagement and the viability of content delivery on breast cancer education through a WhatsApp group. An additional goal was to evaluate the effects of a social interaction channel on key knowledge about breast cancer through WhatsApp. We hypothesized that after 3 weeks of women engagement on the WhatsApp group, knowledge about the risk reduction and early detection of breast cancer would improve.

## Methods

### Study Design

A 3-week pre-post intervention study was conducted among women enrolled in the Brazilian Unified Health System, and a breast cancer screening control study design was adopted to evaluate the feasibility of WhatsApp as an educational tool to increase women's knowledge about the risk reduction and early detection of breast cancer. The study was performed at a basic health unit in the city of Maringá-PR, south Brazil, from July to September 2017. The basic health unit offers free primary care services, including clinical breast examination and mammography, and the annual census reports care for approximately 8000 patients per month. The patient data can be accessed on an electronic information system. The study protocol was approved by the local research ethics committee (protocol number 2.015.313). Written informed consent was obtained from each participant.

### Participants and Recruitment

**Figure 1:**
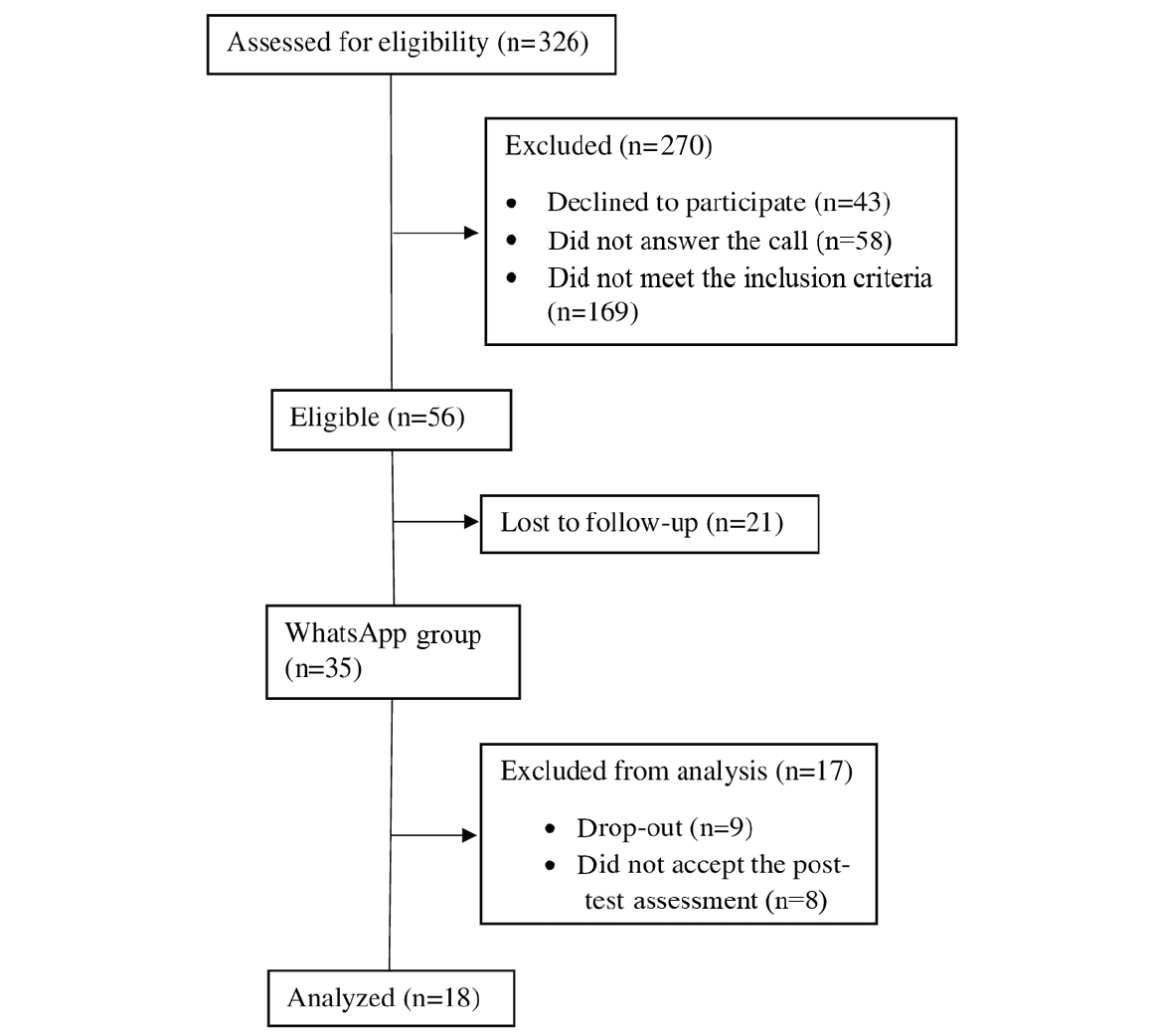
Flow of participants throughout the study.

Participants were adult women aged 45 to 69 years, who were chosen because this is the age group in which the breast cancer incidence is the highest [39]. A sample of 326 women was calculated from a population of 2116 women registered in the information system at the basic health unit. The sample selection process was performed in four steps (Figure 1).

In the first step, stratified randomization was performed by age group. The number of women necessary to compose the sample (*n_g_*) in each regional strata, corresponding to areas of coverage, was calculated according to the following equation:


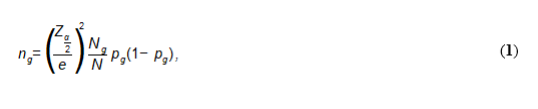


The level of significance considered was α=5%, and the maximum error allowed between the estimate and the real parameter value was e=0.05 (ie, five percentage points). The number of women registered in the *gth* area of coverage is represented by *N_g_*. Additionally, *N* is the total number of women (2116), and *p_g_* is the prevalence of the characteristics to be researched, which was established as 0.5 for all groups, since there is no previous information.

After calculation, the finite population correction factor was used as follows:


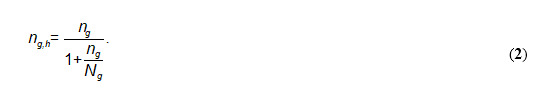


Thus, the sample size obtained with the help of the R Statistical Environment (version 3.2.2, R Core Team) was 326 women.

In the second step, a list was generated from the electronic records of the 326 women in order to identify those who had a cell phone number. After applying this criterion, 110 women were excluded. In the third step, a telephone call was made to the remaining 216 women with the aim to invite them to a face-to-face meeting to explain the study objectives. Among these women, 58 did not answer the calls after three attempts on different days, 45 had incorrect telephone numbers registered, 43 did not show interest in participating in the study, and 14 did not have or use WhatsApp (totaling 160 exclusions). In the fourth step, only 35 of the remaining 56 women attended the meeting, and sociodemographic data were collected and a questionnaire was applied to identify the knowledge level on breast cancer–related topics.

A WhatsApp group, named “AllAboutBreastCancer” (in Portuguese), was created to allocate the 35 women participants, two observers, and one content mediator, who was responsible for sending messages and clarifications. Throughout the intervention, nine women left the WhatsApp group (dropped out of the study) and eight did not answer the questionnaire to identify the knowledge level on breast cancer after 4 weeks of the intervention. Thus, the data of a total of 18 women were analyzed.

### Health Education Content

Topics related to breast cancer were identified on the Brazilian National Cancer Institute (NCI) website*,* and they corresponded to risk factors, protective factors, definitions, incidence, clinical signs and symptoms, diagnostic examinations, mammography, myths and truths, and places to seek support. It is a publicly accessible website. However, both the direct sources of content and the information on the existence of the questionnaire to be used were not disclosed at any time during the intervention.

For each identified topic, we selected specific media (video, audio, or image) and freely available media databases on NCI and Brazilian Ministry of Health websites. In total, two videos, 23 images, and four audios were utilized in the media database.

### Life Habits, Sociodemographic Factors, and Breast Cancer Knowledge

The data were collected at two time points (pre and post intervention) through the application of a two-block structured questionnaire based on information available in the Breast Cancer Early Detection Guidelines, Ministry of Health, and materials from the NCI [40]. In the first block, sociodemographic and life habit characteristics (age, marital status, educational level, religion, and occupation) were collected. In the second block, the knowledge level on breast cancer was assessed, utilizing 18 assertive questions distributed in nine topics as follows: (1) risk factors (questions 1 and 2); (2) protective factors (question 3); (3) definitions (questions 4 and 18); (4) incidence (questions 1 and 4); (5) clinical signs and symptoms (question 5); (6) diagnostic examinations (questions 6-13); (7) mammography (questions 13-15); (8) myths and truths (question 16); and (9) places to seek support (question 17).

The score attributed to each question ranged from 0 to 1, with a maximum score of 18 points. The scores of questions 1, 2, 3, 5, 7, 14, 15, and 16, for which all listed alternatives were correct, were calculated as the number of marked alternatives divided by the total number of alternatives. The score of question 4, for which one alternative was incorrect and the other three alternatives were correct, was calculated as the number of correctly marked alternatives divided by the total number of correct alternatives minus one-third if the incorrect alternative was marked, or was considered to equal 0 if the incorrect alternative was the only one marked. The scores of questions 6, 8, 9, 10, 11, 12, 13, 17, and 18, for which only one listed alternative was correct, was calculated as 1 if the correct alternative was marked or 0 if the incorrect alternative was marked.

### Educational Intervention

The intervention consisted of daily sessions of text, figure, video, and voice messages during 3 weeks. The mediator followed a content submission protocol to send messages between 9 AM and 9 PM. The content was distributed over the period according to the topics. Additional media were created or adapted by the team, according to the demands and dynamics of the group.

### Statistical Analysis

Data were analyzed using the R statistical software (version 3.3.1, R Development Core Team). For the classification of the level of education, we considered the years of education in Brazil, with middle school (minimum 9 years of education), high school (minimum 12 years of education), and higher education (minimum 14 years of education) levels.

To assess the intervention effectiveness, the nonparametric paired Wilcoxon test was applied, with the significance level set at 5%, and the scores obtained before and after the intervention were compared for each individual. The test used is a nonparametric alternative to the paired *t* test when its assumptions are not accepted. In this case, the score distribution was not normal (one of the assumptions), so the Wilcoxon test was chosen.

### Content Analysis

Inductive content analysis [41] was applied for the data extracted from the WhatsApp conversations (exported as a .doc file). The content was converted to an excel file with the following three columns: date/time, user, and message (each message, for analysis purposes, is equivalent to one entry in WhatsApp). The file was imported to be classified and analyzed using NVIVO 11 Pro version (QSR International) for Windows. The messages were initially separated into two categories according to the sender as follows: (1) messages sent by the moderator and (2) messages sent by the users. Emojis were not considered as content to be analyzed.

The preanalysis initially focused on assessment of the 30 most frequent words present only in the user messages, with the goal of identifying the themes that emerged from the group conversations in the original language (Brazilian Portuguese). The parameters used for the preanalysis through the most frequent words in the WhatsApp conversations were as follows: (1) words with four or more characters, (2) words grouped by synonyms, according to the NVIVO 11 Portuguese language dictionary, and (3) exclusion of words that did not have any direct relation to the project content (that, people, during, any, etc). Two of the authors (TFRL and LFG) applied the analysis, and this step focused on building thematic categories that emerged during the process. The following five categories were created: (1) group dynamics; (2) general questions; (3) personal narratives; (4) religious messages; and (5) daily news and events. The data inference, treatment, and interpretation steps focused on the analysis of the text excerpts of each participant within each category, so that they were characterized in the theme. After the categorization process, a translator assisted with the translation of all sentences in each category from Brazilian Portuguese to English.

## Results

### Sociodemographic Characterization and Life Habits

Regarding the sociodemographic characterization of the women who participated in the study, all were over 30 years old, and 9 out of 18 (50%) participants were aged 50 years. For the ethnic-racial profile, 16 out of 18 (89%) participants declared themselves to be white people. Additionally, 10 out of 18 (56%) participants reported a lower degree of education than a completed higher education, 11 out of 18 (61%) declared being married and having children, and 12 out of 18 (67%) declared living with more than two adults. Regarding family income, 6 out of 18 (33%) participants declared monthly income below Brazilian Real 3,520 (approximately US $850). Regarding religious belief, 17 women declared themselves Christian and 1 Buddhist (data not shown).

Concerning life habits, 11 out of 18 (61%) participants reported consuming high-fat foods at least three times a week and consuming fruits and vegetables daily, 15 out of 18 (83%) reported not consuming alcohol or smoking, and 12 out of 18 (67%) reported not practicing physical activity. In addition, 9 out of 18 (50%) participants reported being postmenopausal, 12 out of 18 (67%) reported not using oral contraceptives (however, 11 out of 18 [61%] used them in the past), and 16 out of 18 (89%) reported not taking hormone replacement therapy (however, 12 out of 18 [67%] did take it in the past). Finally, 12 out of 18 (67%) participants did not report any cases of breast cancer in the family and 13 out of 18 (72%) underwent mammography (data not shown).

### Content Analysis

A total of 293 messages were exchanged (moderator 120 and users 173). From the preanalysis step, it was possible to identify five main thematic categories and three subcategories (Table 1).

The category “group dynamics” addressed group participants’ interventions regarding group functioning. In this sense, the following three subcategories stand out in relation to group dynamics: (1) feedback, in which participants express their feelings about the shared content; (2) self-regulation, in which participants express their discomfort regarding content not related to the theme of the group, thus agreeing on some parameters about the group discussions; and (3) coexistence (mostly short messages), in which participants introduce themselves to the group, wish “good morning,” “good afternoon,” or “good night,” and express some expectations about what the group can offer.

The category “general doubts” deals with questions and doubts from the participants mostly about the topic “myths and truths” related to breast cancer. Among the main doubts, there were doubts regarding breastfeeding, contraceptive use, deodorant use, and signs and symptoms that may or may not be related to cancer, as well as lifestyle issues, such as the practice of physical exercise.

The category “personal narratives” deals with participants’ personal reports on their knowledge of cancer cases in their social circle, especially in the family.

The category “religious messages” is about biblical excerpts shared by users without apparent relation to the group work content.

The category “daily news and events” is about the sharing of a public safety event in the city.

**Table 1:** Thematic categories (translated from Portuguese to English).

Topic/description		Illustrative quote
**1. Group dynamics (90 messages)**		
	1.1. Feedback (27 messages)	“I should start doing a sport, lol. Thanks!” “Wow, I’m loving the questions, is clearing up all my doubts” “Hi Antonio, I’m also enjoying it, it’s been very helpful to me” “I’m reading it, liking it and learning, and I’m already talking to my friends to share what I’ve learned” “Very good! Useful information and very important for prevention! I’m passing it along”
	1.2. Self-regulation (35 messages)	“Hi, what’s up? I don’t think it’s cool to start forwarding chain messages in here. The group has a purpose, let’s just talk about it. It’s better this way” “The group’s purpose is to talk about health, I think that is very clear, at least for me” “Girls, I think everything is relevant, but if this group doesn’t stay limited to scientific information about the subject matter, I will leave the group, sorry.” “We have to stay, let’s not make this one slide put all the work in jeopardy. Focus on the discussions”
	1.3. Coexistence (28 messages)	“Good evening, I’m Bethe, we will learn a lot with this project” “Good evening, healthy family! I’m Iracema, it’s an honor to be part of this group, I thank the teachers and academics in acquiring knowledge for a lifetime, taking care of our health. We are here to help each other”
2. General doubts (18 messages)		“I didn’t know that the lack of sport could be one of the causes of breast cancer. Can we say that the intake of pesticides/industrialized products can influence?” “What about the deodorant? I’ve heard rumors that can cause it, is it true?” “Is it true or myth about the breastfeeding?” “True or myth about the use of birth control?” “My daughter with 10 years old had her first period, that concerns me, I heard that when the first period comes the girl stops to grow up, is it myth or true?” “Antonio [moderator], I already had breast cancer and lately I’m overweight, is it true that this is one of the factors that can cause cancer recurrence?” “About the breastfeeding… is there a time for this protection? I breastfed my first child for 5 months and now I’m breastfeeding my second child, but I think I won’t be able to do it for much more than 6 months…” “My sister heard that a spot on the toenail can be a sign of breast cancer. It this correct?” “And the women with less than 50 years old? Can or should do the mammogram?” “Good evening, Antonio. My daughter took the first dose of HPV vaccine with 10 years old, the second dose would be after 6 months and she hasn’t taken for 10 months now, can she still take the second dose?”
3. Personal narratives (7 messages)		“I also have cases in my family, my sister decided not to have children and had breast cancer, she found out in the beginning and treated it… but she travels to Sao Paulo every 6 months to have a follow-up care!” “My mother-in-law had breast cancer 3 years ago, and now has been diagnosed with liver and lung metastasis.” “My maternal grandmother had it. Today she is with 94 years old, this was 20 years ago.” “My mother had it with 45 years old. It’s been 17 years.”
4. Religious messages (3 messages)		“What are the commandments? Asked the young man. Jesus said to him, “Do not kill, do not commit adultery, do not steal.” “Do not bear false witness. Honor Father and Mother. Love your neighbor as yourself. Matthew 19, 16-22”
5. Daily news and events (1 message)		“Everyone, I’m sorry to send this message right now, but almost 90 prisoners escaped the Maringá prison. It broadcasting in media news alerting residents and people in towns nearby. Be very careful. #scary”

### Breast Cancer Knowledge

**Figure 2:**
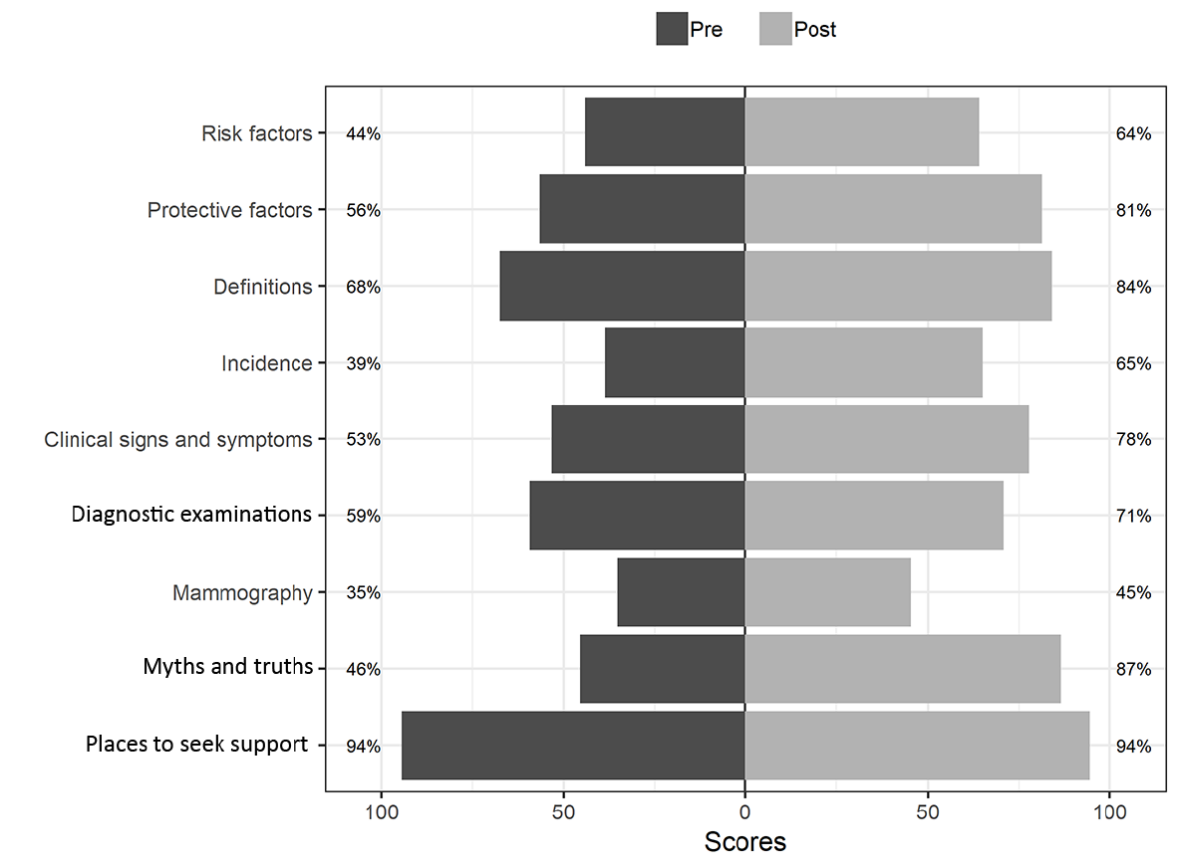
Profile of scores obtained in each domain of the questionnaire designed to analyze the level of knowledge about breast cancer.

Figure 2 presents data regarding the scores obtained by participants in the nine domains of the questionnaire that was designed to analyze the level of knowledge on breast cancer and was applied before and after the educational intervention. The topics presenting a lower score gain in the posttest analysis were “definitions” (16 points gained), “examinations” (12 points gained), and “mammography” (10 points gained), whereas the topics with the highest gains were “myths and truths” (41 points gained), “incidence” (26 points gained), “clinical manifestations” (25 points gained), and “protective factors” (25 points gained). It was observed that beside “places to seek support,” which remained stable, there were score gains in all 80 domains or topics of the questionnaire in the pre-post intervention.

Regarding factors that increase the chances of developing breast cancer, before the intervention, 9 out of 18 participants mentioned only family history of breast cancer, smoking, and alcohol consumption. After the intervention, all listed factors had a higher frequency of response, and factors, such as consumption of high-fat foods, first pregnancy after 30 years of age, first period before 12 years of age, and end of menstruation after 55 years of age, were mentioned by less than half of the women.

Regarding family history, before the intervention, 15 out of 18 participants indicated that having at least one first-degree relative diagnosed with cancer in one breast increases the risk of developing this type of cancer in women under the age of 50 years, which did not change after the intervention. Similarly, most of the participants responded that the risk also includes a family history of at least one first-degree relative diagnosed with cancer in both breasts or ovarian cancer in any age group, which also did not change after the intervention. The increased risk for family history of male breast cancer was only mentioned after the educational intervention by one-third of the participants.

Concerning protective factors, before the intervention, 15 out of 18 participants mentioned only diet with many fruits and vegetables, which did not change after the intervention. However, all the other listed protective factors had a higher mentioning frequency after the educational intervention, with breastfeeding as the most frequently mentioned protective factor (16 out of 18 participants).

Regarding knowledge about the concept of the disease, before the intervention, 8 out of 18 participants incorrectly pointed out that breast cancer can be a benign tumor. After the intervention, this answer was chosen by only three participants. The most mentioned signs and symptoms before the intervention were small nodules in the underarm (armpit) or neck region and the appearance of a fixed usually painless lump, which did not change after the educational intervention.

Regarding the early detection of breast cancer, before the intervention, the three recommendations listed as ways of contributing to early detection were mentioned by 9 out of 18 (50%) participants, with mammography as the main examination for lesion tracking. After the intervention, all of these items were mentioned more frequently. In addition, after the intervention, all of the participants stated that doing the breast self-examination or being asymptomatic does not exclude the necessity for a clinical breast examination or mammography.

Nevertheless, after the intervention, most women indicated the correct concept of clinical breast examination and pointed out the recommendation of age range and periodicity for the examination. Lastly, it was observed that all the participants continued to point out that the basic health unit was an ideal place to look for assistance and information regarding doubts or any initial manifestations. After the intervention, all women mentioned “breast cancer is curable.”

Regarding mammography, most participants had already scored correctly in the examination concept, maintaining the score after the intervention. However, the same was not observed with the recommendations regarding periodicity, age group, and details about the examination, as most women did not score correctly.

After the intervention, all the listed statements were considered as myths with more frequency, and the most mentioned was “large breasts represent a higher risk of developing the disease.”

**Figure 3:**
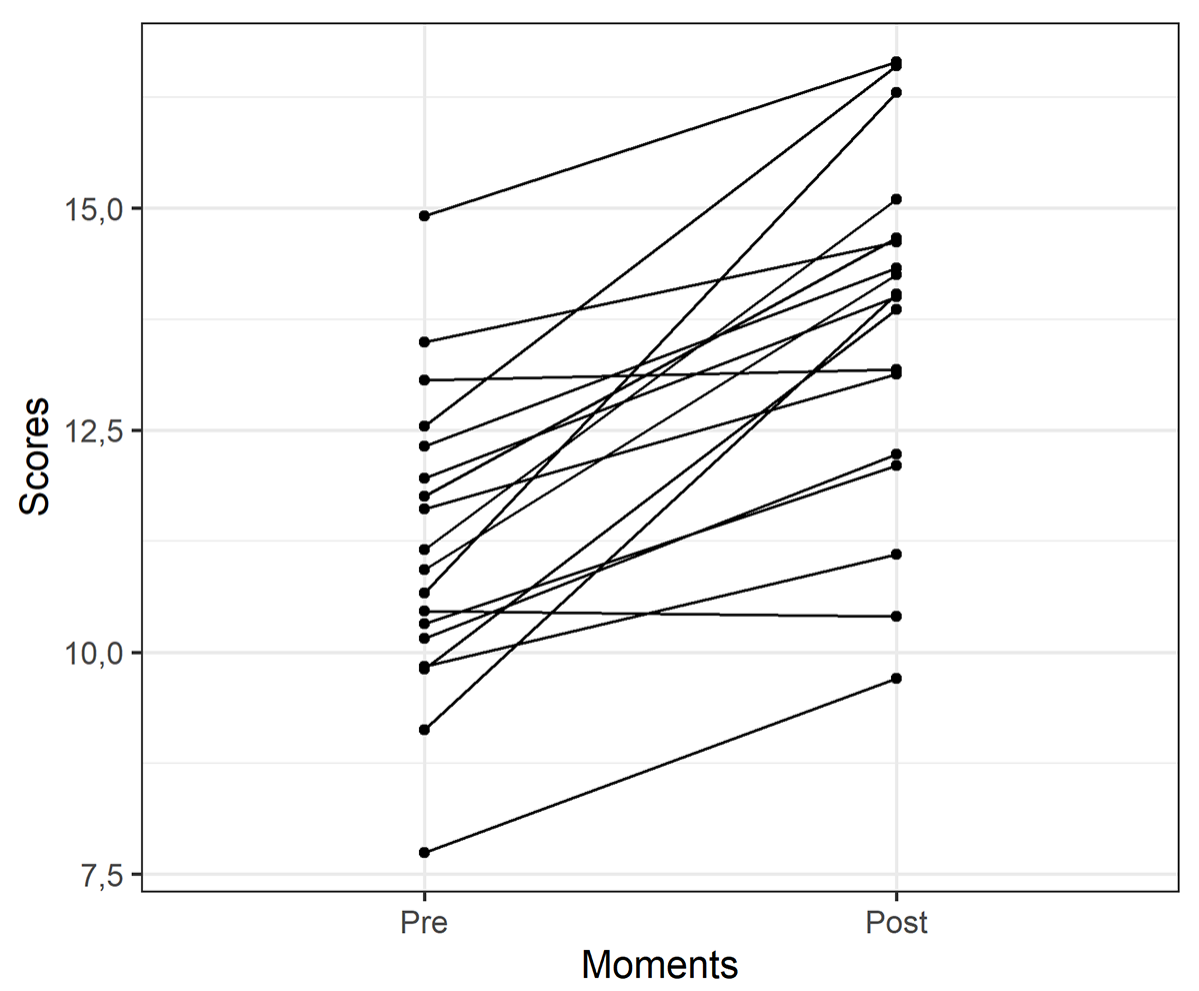
Profile of individual scores obtained in the nine domains of the questionnaire designed to analyze the level of knowledge about breast cancer.

Individual data regarding the effect of the educational intervention on improving knowledge about breast cancer are shown in Figure 3. The score regarding the knowledge of breast cancer tended to increase from before to after the educational intervention for each study participant. The average scores of the participants were 11.21 and 13.68 points before and after the intervention, respectively, and there was sufficient sample evidence that the difference was significant, according to the paired Wilcoxon test results, with a significance level of 5% (*P*<.001) (Table 2).

**Table 2:** Descriptive measures of the scores obtained in the nine domains of the questionnaire designed to analyze the level of knowledge on breast cancer applied before and after the educational intervention, according to the paired Wilcoxon test.­

Moment	Mean	SD	Median	Q_1_-Q_3_	*P* value
Preintervention	11.21	1.70	11.04	10.20-12.23	<.001
Postintervention	13.68	1.99	14.02	12.46-14.65	

## Discussion

### Principal Findings

To our knowledge, this is the first study to use WhatsApp as a health education tool for Brazilian women from the public health system, with a focus on the risk reduction and early detection of breast cancer. The 3-week intervention, which used the app as an online space to mediate content selected from the NCI and the Brazilian Ministry of Health websites, enabled women to improve their knowledge in all topics addressed, especially in “myths and truths,” “incidence,” “clinical manifestations,” and “protective factors.” The app, which is known by women as a daily tool to interact with friends and relatives, allowed a receptive space to solve doubts not commonly discussed during presential care. In general, the results obtained herein suggest that WhatsApp could be incorporated as an additional tool in traditional health communication actions and campaigns.

### Use of WhatsApp

No woman reported having difficulties when interacting with the device or app, or asked for training on how to use it. Many aspects may have contributed to the gains in pre- and postintervention scores. The combination of text, image, video, and audio may partly explain the gain in knowledge. This multimedia aspect differentiates WhatsApp interventions from other mobile interventions that exclusively use SMS text messaging. Mayer [42] has talked about “multimedia learning” and has argued that well-designed multimedia content (the combination of video, audio, and text) can enhance the learning process when compared with an educational process that uses only one modality (eg, only text). In addition, the possibility to send messages directly to each woman has been highlighted as a positive aspect, since some messages were designed for each doubt. Both health communication [43] and neuroscience researchers [44] have demonstrated that tailoring health behavior intervention materials increases the effectiveness of health messages.

The data confirm the viability of the app as a health learning tool. However, the majority of articles that describe this type of gain involve health students or health professionals [28]. For example, Khanna et al [45] used the app to optimize communication within a group of residents in the orthopedic sector. Graziano et al [46] used it for neurosurgeons in Italy, and Gulacti et al [47] used it in the emergency sector. Johnston et al [48] applied the app effectively among health professionals. Regarding patients and professionals, the app is described as a communication channel, but is not linked to an educational process, as observed in the report by Petruzzi and De Benedittis [49] (use of WhatsApp for oral health care) and by Krynski et al [50] (pediatricians and patients in Argentina). However, data exchange or tele-consultation is still a matter of debate, since messages exchanged in commercial apps represent a risk for data that should be more confidential [51]. According to Watson et al [52], in response to Johnston et al [48], even after the adoption of encryption technology, the app does not meet the standards required for clinical data transfer, and consequently, it is not recommended for use in a clinical environment. Even as an education and intervention tool, which was the case in this study, we understand that the app is a commercial platform with financial interests and that all interactions are converted into data to analyze user behavior patterns, which can be used in digital marketing strategies. Facebook Inc, the owner of the app, leads this commercial usage.

Regarding the advantages and disadvantages of the use of WhatsApp in health care settings, two positions seem to coexist in the scientific debate. First, one that exposes and underlines all of the positive aspects of the phenomenon as follows: improvement of communication, no requirement for a computer, time saving, possibility of an immediate response, improvement of health worker performance, reduction of consultation time, and smoothening of hierarchy between health groups. Second, one that highlights the negative aspects linked in particular to the clinical risks for patients, data security, and privacy protection [53].

In another promising direction, the app was used in social marketing campaigns to increase the uptake of free mammography for underprivileged women in Malaysia [54].

When compared with other types of media, the advantage of using this timeless tool is always mentioned. Web-based forums on websites are alternatives to debate about breast cancer [55,56], and even online social networks, such as Facebook, have been used before as an educational channel between women and health workers [57,58]. However, WhatsApp is a more pervasive, ubiquitous, and ready-to-hand tool than forums or websites. Before the popularity of this app, previous research in Brazil pointed out that television was the most used source by interviewees to acquire knowledge about breast cancer [59]. In 2018, WhatsApp was mentioned as a communication channel that 66.5% of women with cancer in Ecuador would like to use to receive information about the pathology [17].

In our case, the group dynamics and feedback shared by the users confirmed that the app is an informal environment of learning. During the intervention process, three women mentioned that they added value to the content and shared the knowledge and media to friends and relatives. This easy possibility to share content with the tool can also extend the impact of our intervention beyond the intended primary target [60]. The participants made the following comments:

I’m reading it, liking it and learning, and I’m already talking to my friends to share what I’ve learned.

…I’m passing it on

…Participating in this project was really good, besides from adding knowledge I also pass it on a lot to my relatives, Antonio.

In addition to the informal environment, the diversity of women and the quality of grouping them (since they were not a group before the intervention) may have contributed to some conflicts during the intervention (see coexistence and self-regulation messages). There were 35 messages dedicated to self-regulation, where the participants negotiated what kind of content can be shared. Some of the messages were as follows:

Hi, what’s up? I don’t think it’s cool to start forwarding chain messages in here. The group has a purpose, let’s just talk about it. It’s better this way 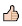

Girls, I think everything is relevant, but if this group doesn’t stay limited to scientific information about the subject matter, I will leave the group, sorry.

The chain messages are good, but let’s focus on our health.

Deviation of the main theme is a natural phenomenon from a diverse group, and the dos and don’ts inside the community were expected to avoid information overload. However, WhatsApp allows copying and forwarding messages of other groups, which has been described by Church and De Oliveira [61] and was perceived as a problem between some users in our educational intervention using the app.

During the intervention process, the creation of a friendly and harmonious interaction environment (a persistence online space) enabled the users to continually handle situations and slowly establish social norms and community parameters. They decided what could and could not be shared between them. In this case, the qualitative data showed two topics that emerged during the intervention, which were not directly related to the initial planned themes, as follows: (1) religious messages and (2) daily news. These deviation topics triggered a heated regulation discussion, causing the drop-out of three women from the group (nine during all the intervention processes). Although they belonged to the same neighborhood, the fact that the intervention was preventive was not enough to create a sense of identity as in studies involving patients already with the disease. It is relevant to remember that researchers who deal with online social networking emphasize that groups are assembled by common interests and not by geographic location [62,63]. Likewise, in the first face-to-face meeting, we observed that the initial moment in the intervention (when participants introduce themselves) was not able to connect them, and just like in-person groups, the social ties in an online environment also need interaction time to be effective [64].

In the first case of one of the themes shared among the participants (religious messages), the literature shows that cancer is usually and symbolically associated with death and religious topics [65]. Indeed, religious faith is praised as a provider of hope, optimism, and empowerment for diagnosed patients [66]. However, in our intervention, this topic was not related to breast cancer. An entire Bible quotation was sent in the group (cut-and-paste content of over 3000 characters), and some of the users did not enjoy the content or even found it boring. Another religious video was identified as fake (voice over the original content) by one of the women.

The other topic “daily news” occurred after the news “prisoners that escaped the Maringá prison.” The concern about security was shared in the group, advising others to stay safe at home. For an alert (in the sense of “breaking news”), WhatsApp was perceived as a better tool to share information, overpassing the primary goal of the group.

### Mammography

We believe that the topics “definitions,” “examinations,” and “mammography” corresponded to the content with the lowest score gain because they are of technical or instrumental nature and require more cognitive resources to memorize concepts, such as age range, conceptual differences, and high-risk criteria. A study performed with 914 women also identified limited knowledge about mammography [67]. The diversity of access to information about examinations disseminated by media could pass on information that may cause confusion among recipients, clarifying the central theme without making it easier to understand the related issues [67].

In our intervention, information was delivered with emphasis on the recommended age range for the examination and its technical characteristics. Exposure to breast cancer content traditionally puts women in a passive position [68]. Thus, the low gain in knowledge on this topic can be explained by the way we approached it, without rescuing the mammography experience of the participants [69].

In addition, a study analyzing the online search behavior of women on mammography identified that there is a greater interest during the month in which the national campaign called “Pink October” occurs every year [70]. In our case, WhatsApp enabled timeless contact about the theme, thus extending the talk about the disease beyond “Pink October.”

### Myths

Myths and truths about cancer received more interaction and more questions, and similarly, it was the topic that showed gain in the posttest period. We understand that health professionals preferably provide medical information to avoid misinformation. In our case, the tutor was responsible for managing the content by provoking discussions about it.

We did not find any studies that discussed women’s knowledge gain related to the deconstruction of popular untruths common in daily life. Given the frequency at which these issues are debated among laypeople and the impact on the behaviors of individuals, such as wearing a bra, using deodorant, and using breast prosthesis, it is assumed that the level of interest and attention to this topic was a variable that contributed to the gain.

The doubts formulated by the participants can be compared as “fake news” contents, a key topic of discussion nowadays, which is alarming public health authorities because of its potential to spread quickly [71] and the requirement of an approach to counteract such misinformation [72]. WhatsApp has been accused of being a protagonist channel of misinformation, including during the presidential elections in Brazil [73]. Doubts revolved around topics seeking to understand breastfeeding, deodorant use, human papillomavirus vaccine, and obesity. However, some questions were straightforward as follows:

Can you say that the intake of pesticides/industrialized products can also influence?

Additionally, there were concerns about protective factors as follows:

About the breastfeeding… is there a time for this protection? I breastfed my first child for 5 months and now I’m breastfeeding my second child, but I think I won’t be able to do it for much more than 6 months...‬

### Sharing Personal Narratives

The cancer theme is indeed very complex. Doubts indicate the need to increase the health literacy of women, and even with the investments in health communication strategies during the month of the campaign “Pink October,” frequent doubts remain.

“Pink October” is a set of communication strategies integrated by the NCI since 2010 as part of the National Breast Cancer Control Program, with adherence to several television programs. However, despite the national effort, it was noted in a study from 2013 that the campaign was able to legitimate an audience (people were able to interpret and judge the message conveyed as positive and important), but was not effective for behavior change [74]. It is worth remembering that the increase in the number of cancer cases is due to a lack of regularity in prevention campaigns. Additionally, the fact that campaigns do not reach the entire country and the difficulties of representing and demystifying cancer as a fatal disease have been mentioned [68].

A positive moment of interaction was when participants shared family history of cancer. The narratives and descriptions were an opportunity for the mediator to later make some comments about risk factors. The stories were about close relatives as follows:

I also have cases in my family, my sister decided not to have children and had breast cancer, she found out in the beginning and treated it…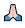 but she travels to Sao Paulo every 6 months to have a follow-up care!

Even stories concerning people with no direct genetically bonds were shared as follows:

My mother-in-law had breast cancer 3 years ago, and has now been diagnosed with liver and lung metastasis 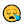.

Another woman recognized the risk factor by considering the genetical factor as follows:

I don’t have any genetic factors or cases in the family, but I am already 55 years old and very sedentary. Take some action, walk! 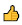.

Additionally, previous stories of survival were shared as follows:

My maternal grandmother had it. Today she is 94 years old, this was 20 years ago.

My mother had it with 45 years old. It’s been 17 years.

No death stories were shared. This aspect may have been influenced by the usage context of WhatsApp, which is seen by many users as more informal when compared with SMS text messaging. In a study by Church and De Oliveira [61], users reported that the tool allows a more fluid and open conversation.

Regarding the protective factors, before the intervention, 15 out 18 (83%) women only mentioned eating many fruits and vegetables, which did not change after the intervention. However, all other listed protective factors had a higher frequency of occurrence after the educational intervention, with breastfeeding being the most frequently mentioned protective factor (16/18, 89%). Not coincidentally, the participants formulated many interactions and questions about breastfeeding as a protective factor during the intervention.

Even with formulated doubts, the participants waited for expert clarification. None of the women in the group tried to solve the doubts or answer them. A more dialogical approach, in which women participate more and do not wait for an “expert voice,” can add another perspective. Our intervention may have reinforced a receptive profile to messages rather than the creation of an active approach, recognizing the role in the search of information. These challenges had already been identified in previous group research, which pointed out that women preferred to expose themselves to content already created by health authorities (particularly in the figure of a doctor). This can be the result of the following two aspects: (1) women did not feel as part of a group or had the willingness to collaborate and try to find answers for themselves and (2) there is a passive posture for receiving information about cancer.

Despite the “Pink October” campaign in the country, breast cancer still represents a challenge for the public health sector, which needs more efficient screening techniques for early diagnosis. Thus, communication challenges and health education actions encourage alternative communication tools that add up to traditional campaigns. The use of WhatsApp is promising because it is a low investment tool and can act on a massive scale, especially in developing countries, such as Brazil, where there are higher rates, mainly due to the lack of early detection and adequate treatment [75].

### Limitations

The authors acknowledge that the mediation that directed the content may have contributed to the maintenance of a passive posture by the participants on receiving messages. However, during the intervention, owing to the high necessity of the mediator to suggest and deliver content, we perceived a challenge to provide a top-down model of health information release and to place individuals as participants in the teaching process and health learning approach.

In addition, we recognize that a more expressive sample is important to perform a more meaningful analysis. Our initial analysis was based on a representative sample size of the target population of the study. However, participant loss was greater than expected. The reasons were diverse but consistent with the peculiarities of the investigated population. Nevertheless, we believe it is relevant to present the results of the statistical analysis, as they enable a better understanding of the phenomena that occurred throughout the intervention when integrated with the qualitative analysis.

Lastly, we highlight that even though knowledge gain can create a change in behavior, this is a multifactorial aspect of complex measurement. In our initial proposal, there was a desire to follow the participants in order to observe if the group would continue with new behaviors to reduce the risk of breast cancer after the end of the study. However, owing to the high dropout rate at the beginning of the study and recruitment difficulties, we chose to implement the analysis of knowledge gain.

### Conclusions

This study explored the use of WhatsApp as a tool to facilitate knowledge exchange for the risk reduction and early detection of breast cancer between women and a content moderator. The results confirm our hypothesis that the app is a useful tool that can be incorporated into health education strategies that focus on breast cancer. Although the intervention improved the knowledge of the participants with regard to breast cancer, they expected that the moderator would send content and solve their doubts, suggesting a need for new strategies directed at the encouragement of dialogic communication. Nevertheless, the results obtained demonstrate that WhatsApp is a feasible online space for women to seek answers for general doubts that are not covered in communication campaigns on breast cancer.

## Abbreviations

NCI: (Brazilian) National Cancer Institute
